# Bell County Medical Society

**Published:** 1903-10

**Authors:** 


### Bell County Medical Society.

TEMPLE, TEXAS, October 5, 1903.

*Texas Medical Journal:*

The Bell county doctors met at Belton, October 3rd, and organized the Bell County Medical Society, according to by-laws and constitution of American Medical Association, with thirty-nine charter members.

J. M. Frazier, M. D., Belton, President; J. H. Payne, M. D., Holland, Vice-President; J. M. McCutchan, M. D., Temple, Secretary.

---

COLORADO, TEXAS, October 5, 1903.

*Editor Texas Medical Journal, Austin, Texas:*

The counties of District No. 2, so far as organized, are as follows:

September 1st, the Nolan-Fisher-Stonewall Counties Society was organized with twelve members. President, Dr. A. M. Davidson, of Roby; Secretary, Dr. J. R. Roebuck, of Sweetwater.

September 12th. Taylor County; fifteen members. President, Dr. T. D. Bass, Abilene; Secretary, Dr. J. B. Thomas, Abilene.

September 26th. Mitchell-Scurry-Kent-Dickens Counties Society; twelve members. President, Dr. W. H. Morrow, Dunn, Scurry county; Secretary, Dr. W. R. Smith, Colorado.

September 30th. Jones-Haskell-Knox-King Counties Society; sixteen members. President, Dr. R. R. Shappard, Anson; Secretary, Dr. Lee Williams, Anson.

The plan of organization is heartily endorsed by the physicians of this section, and the enthusiasm with which they are entering into the work is very gratifying to the councilor.

Showing importance of organization, it was found only five out of the fifty-five in societies so far organized belonged to the State Association. The remainder of the list will be completed at an early date.

Very truly yours,

P. C. COLEMAN, M. D.,

Councilor, District No. 2.

[Dr. J. R. Roebuck, Secretary of the Nolan-Fisher-Stonewall Counties Medical Society, writes us that the next meeting of that society will be held at Sweetwater, the first Tuesday in December. —ED.]

